# A sobrevivência da Antiguidade nas capas e propagandas de medicamentos da revista Eu Sei Tudo, 1917-1958^*^

**DOI:** 10.1590/S0104-59702026000100004

**Published:** 2026-03-30

**Authors:** Andréa Casa Nova Maia, Douglas de Souza Liborio

**Affiliations:** i Professora, Instituto de História/Universidade Federal do Rio de Janeiro. Rio de Janeiro – RJ – Brasil andreacn.bh@gmail.com; ii Doutorando, Programa de Pós-graduação em História/Universidade Federal Fluminense. Niterói – RJ – Brasil. douglasdesouzaliborio@gmail.com

**Keywords:** Cultura visual, Antiguidade, Corpo, Indústria farmacêutica, Brasil republicano, Visual culture, Antiquity, Body, Pharmaceutical industry, Republican Brazil

## Abstract

O artigo propõe uma análise de imagens de capas e propagandas de medicamentos da Bayer e da Vikelp no intuito de problematizar a sobrevivência da Antiguidade greco-romana na revista ilustrada *Eu Sei Tudo*, que circulou no Brasil entre 1917 e 1958. A análise privilegiou referências teórico-metodológicas e conceitos produzidos por Aby Warburg em suas análises sobre as ninfas e o trabalho de Georges Didi-Huberman, que também pensa a imagem sobrevivente. Problematizamos a presença das divindades na revista, com foco na discussão sobre os ideais de beleza, perfeição e força propagados pela indústria farmacêutica, bem como a saúde e o corpo saudável, que se apresentam na modernidade atravessados pelas referências greco-romanas.

## Abstract (en)

This article explores images from the covers and advertisements for drugs from Bayer and Vikelp in an effort to investigate the survival of Greco-Roman antiquity in the illustrated magazine Eu Sei Tudo, which circulated in Brazil from 1917 to 1958. The analysis utilizes theoretical and methodological references and concepts from Aby Warburg in his analyses on nymphs and Georges Didi-Huberman, who also considered the surviving imagery. We question the presence of divine characters in the magazine, with a focus on the ideals of beauty, perfection and strength propagated by the pharmaceutical industry, as well as Greco-Roman references in modern representations of health and the healthy body.

*Eu Sei Tudo* foi lançada no Brasil em 1917 e permaneceu até 1958. Publicada pela Companhia Editora Americana, no Rio de Janeiro, tinha como proposta ser uma revista mensal ilustrada, destinada a oferecer ao público uma variedade de conteúdos que ia de artigos científicos a literários, históricos e artísticos. Sua abordagem refletia as transformações culturais da então capital federal, integrando-se ao ambiente urbano carioca e atendendo aos interesses de uma classe média em expansão. O modelo editorial foi inspirado na revista francesa *Je Sais Tout*, idealizada por Lafitte em 1905, que circulou na França até 1939. Esse periódico europeu foi pioneiro em combinar o formato de uma enciclopédia popular com o apelo visual de ilustrações atraentes, embora tenha enfrentado interrupções durante a Primeira Guerra Mundial e, décadas depois, uma breve retomada pela Hachette, em 1969 e 1970, com um novo projeto gráfico (Maia, 2014).

*Eu Sei Tudo* se consolidou como uma publicação de referência, valorizada por sua diversidade temática e por seu rico formato. Seu *layout* seguia o padrão de grandes almanaques da época: aproximadamente 150 páginas, dimensões de 26,5cm por 17,5cm, encadernação em brochura e papel de qualidade superior. Algumas páginas traziam ilustrações coloridas, enquanto outras exploravam o preto e branco ou tonalidades bicromáticas. Comercializada por meio da venda direta em bancas ou por assinaturas, o custo inicial da revista era de 2$000, subindo para 2$200 com o passar dos anos. Para comparação, esses valores permitiam comprar itens como manteiga (2$300) ou uma dúzia de ovos (1$000). Paradoxalmente, a moderna revista trazia em suas páginas referências às mitologias da Antiguidade, não só em suas capas, mas no projeto gráfico e em propagandas de remédios. O artigo procura demonstrar como a sobrevivência das imagens antigas na revista ilustrada integra a cultura visual da vida moderna, simbolizando força, beleza e poder para os corpos dos consumidores de fármacos como Bayer e Vikelp.

## A sobrevivência da Antiguidade nas capas da *Eu Sei Tudo*

Identificamos um expressivo *corpus* iconográfico com referências à Antiguidade greco-romana, numa relação singular com o conteúdo e o programa gráfico da revista. A ampla gama de imagens produzidas no Brasil na primeira metade do século XX na *Eu Sei Tudo* integrou um peculiar processo de modernização artística oriundo da cultura midiática urbana do Rio de Janeiro. Cardoso (2022, p.145-147) aponta como os impressos ilustrados que afluíram no cenário carioca do início da República propiciaram possibilidades inéditas de experimentação: a produção gráfica das revistas promovia uma cultura visual vibrante que permitia flexibilidade na combinação de modelos de um determinado tempo e espaço com a realidade brasileira.^1^

Uma das características da *Eu Sei Tudo* era justamente a grande quantidade de propagandas que se dedicava a constituir o imaginário do “viver moderno” que emergiu no mundo ocidental no início do século XX: artigos de moda, produtos para o lar e cuidado com a saúde eram constantemente veiculados a partir de associações com mitos e divindades pagãs, em grande parte, femininas (Maia, Oliveira, 2020). Podemos dizer que era quase uma tônica da revista, pois a identidade da mulher “moderna” representada com atributos da Antiguidade era recorrente nas suas páginas. Na capa havia experimentações arrojadas de ilustrações e fotografias criando efeitos cromáticos inovadores visando a um determinado efeito decorativo, algumas vezes assinada na parte inferior pelo artista responsável.

**Figura 1:**
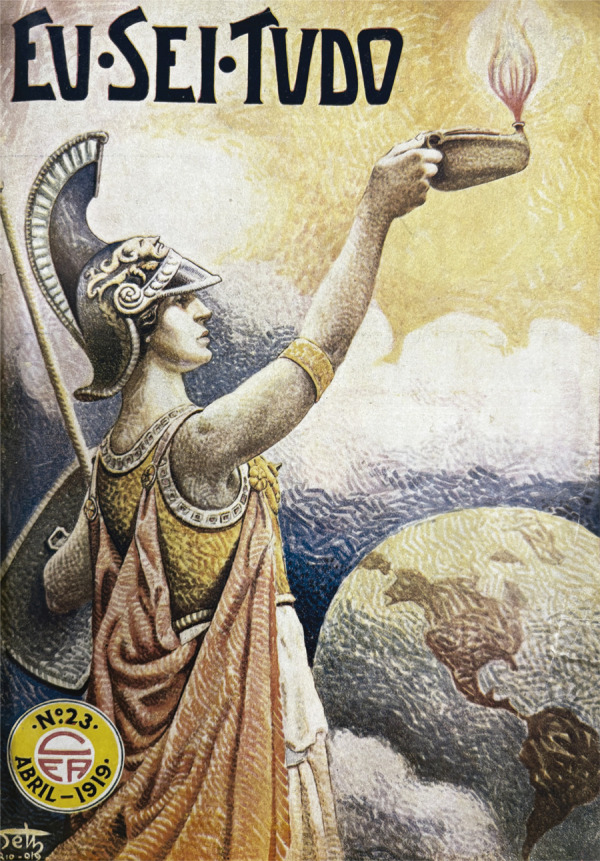
: Capa do número 23 da revista *Eu Sei Tudo*, ilustração de Seth (Eu Sei Tudo..., abr. 1919)

Destacamos aqui três capas da *Eu Sei Tudo* que selecionaram e adaptaram formas e figuras da Antiguidade greco-romana. A primeira data de abril de 1919: a ilustração é assinada por Seth, pseudônimo do desenhista Álvaro Marins (1891-1949), mestre do bico de pena e da produção de caricaturas aquareladas para as revistas ilustradas da época (Lima, 1963).^2^ Seth nos apresenta a figura imponente de uma mulher trajada à antiga, tendo ao fundo o globo terrestre com destaque para o continente americano. Em pose altiva, é representada com atributos típicos da deusa Minerva: na mão esquerda, porta a lança e o escudo; na cabeça, o capacete que caracteriza sua vocação para a guerra e a paz, com a imagem da Górgona que compõe a iconografia da deusa (Ménard, 1985, p.193-194). Com a mão direita, porém, ela ergue uma lucerna acesa: a lâmpada a óleo com o fogo é tradicionalmente um atributo das virgens vestais na Antiguidade romana. As vestais eram sacerdotisas consagradas à deusa Vesta,^3^ encarregadas de velar o fogo sagrado perpétuo do altar da cidade, que representava a vitalidade da República (Brandão, 2009, p.291-292) (Figura 1).

A capa demonstra como o repertório visual das divindades femininas pagãs sobreviveu até a modernidade, tendo sido ressignificado na criação do imaginário político dos Estados nacionais. O uso da referência mitológica para a representação da mulher como uma deusa cívica é compreendido ao remeter a outras imagens da *Eu Sei Tudo* no mesmo período: o retorno à Minerva também foi mobilizado para as narrativas de vitória e pacificação e constituídos no período entreguerras (1918-1939). Na expressão da guerra justa e da estabilidade republicana, a imbricação das divindades cria uma cenografia a partir de um passado recuperado e articulado não como nostalgia romântica, mas reinventado num amplo cenário alegórico atravessado por uma temporalidade multidirecional (Didi-Huberman, 2019, p.278).

**Figura 2:**
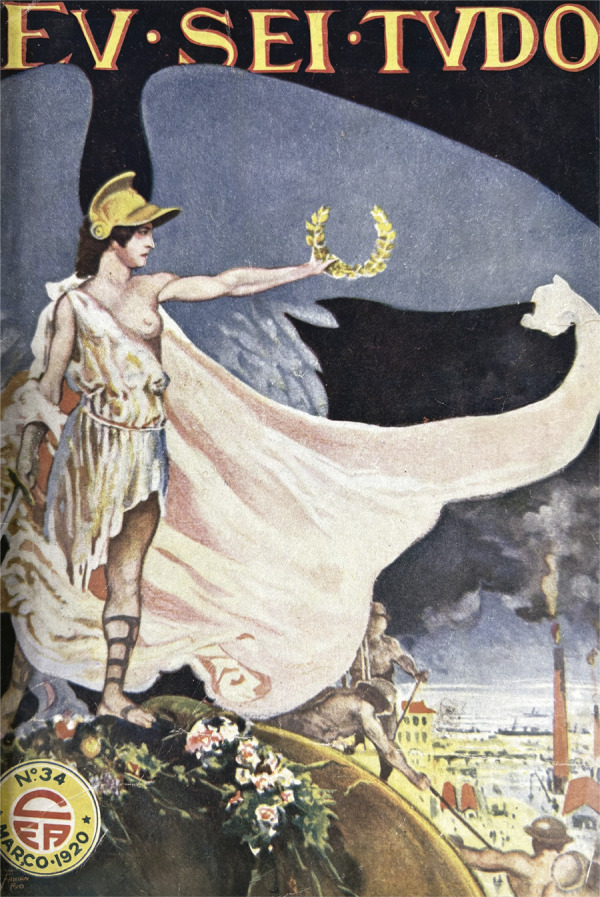
: Capa do número 34 da revista *Eu Sei Tudo* (Eu Sei Tudo..., mar. 1920)

Assim, podemos entender a revista como centro nevrálgico de atravessamentos, com enorme potencial de evocação e colisão de tempos, ideias e imagens, criando algo novo, onde o retorno ao antigo detém expressivo potencial visual. Essa potência a partir de elementos “antiquizantes” dão o sentido das narrativas cívicas: a capa da *Eu Sei Tudo* de março de 1920 apresenta a imagem de uma deusa guerreira alada com destaque sobre o globo terrestre, a espada na mão direita e uma coroa de louros na esquerda, descortinando-se um cenário fabril. Na gestualidade e no movimento da capa, nas asas e no ato de erguer os louros subsiste a referência a outra figura do panteão romano: a deusa Victoria, imagem que perdurou na arte ocidental sendo utilizada para personificar triunfos (Figura 2).^4^

Nas capas, ora a pátria guerreira, ora a mulher moderna, como na edição de março de 1930: a protagonista, no desenho de Gene Pressler, é uma representação graciosa da mulher renovada que despontava num Brasil que buscava consolidar uma imagem cosmopolita. O ilustrador, famoso por suas mulheres estilizadas e idealizadas, apresenta uma alegoria sofisticada, com cabelo *à la garçonne* e boca em sorriso vaidoso, corpo solto em movimento com um apelo da graciosidade e elegância pela exibição de um longo traje. A imagem da moda e da feminilidade se constrói pela própria indumentária e na cenografia da capa: a cauda do tecido ornamentado acaba fundida com a plumagem de um alvíssimo pavão, como era típico das ilustrações do ilustrador novaiorquino.

**Figura 3:**
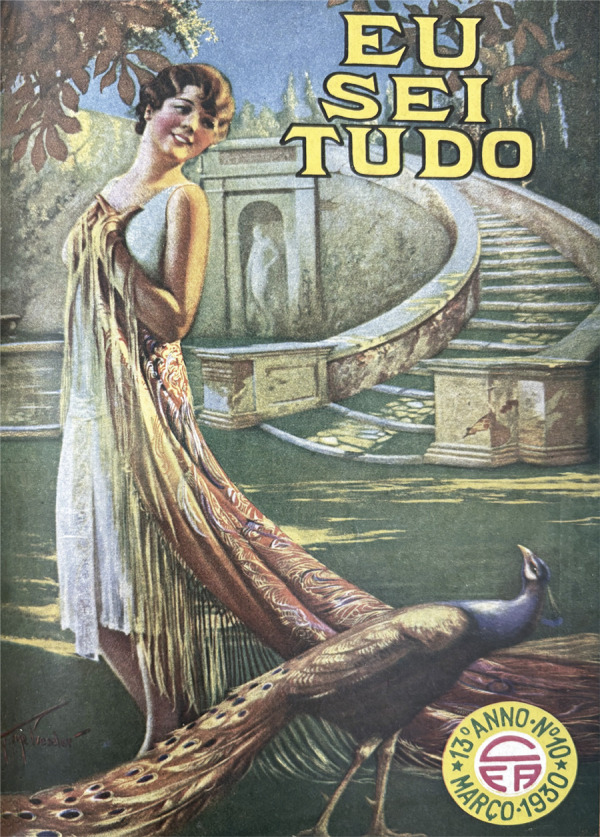
: Capa do número 154 da revista *Eu Sei Tudo* (Eu Sei Tudo..., mar. 1930)

Na mitologia, o pavão é o animal sagrado da deusa Juno, divindade feminina esposa de Júpiter (associado ao Zeus grego). Costumeiramente aproximada à imagem da Hera dos gregos, Juno faz parte da Tríade Capitolina romana, sendo considerada a deusa da fecundidade e deusa rainha. Simboliza o princípio feminino por excelência, na sua maturidade, sendo a protetora das mulheres casadas e dos nascimentos legítimos (Chevalier, Gheerbrant, 2020, p.591-592). Na capa do número 10 da revista, sua representação é emoldurada por escadaria que abriga um nicho com escultura em mármore em pose sinuosa similar à da mulher (Figura 3).

Nas três capas, percebemos um eco de outra temporalidade emergindo pelos entrelugares da composição, entre o moderno e o antigo. De Minerva a Juno, entrevemos a imagem sobrevivente construindo sentidos para o olhar de quem prende sua atenção por algum tempo na capa, ansiando por desbravar um pouco desse panteão em cada página.

## Alguns apontamentos teóricos sobre a sobrevivência da Antiguidade na revista

A partir da apresentação geral desse aspecto da *Eu Sei Tudo*, impõe-se a seguinte questão: qual seria o sentido da inserção de figuras mitológicas para a expressão de determinadas formas de comportamento para a vida moderna brasileira? Segundo Silva (2007, p.30) deve-se empreender uma leitura mais discursiva do mundo antigo, fugindo à linearidade tradicional, “apontando sempre para uma Antiguidade guiada, imaginada, reconstruída” num dado contexto.

Concordamos com a argumentação de Didi-Huberman (2013, p.66) ao considerar a Antiguidade “um grande movimento de terrenos, uma vibração surda, uma harmonia que atravessa todas as camadas históricas e todos os níveis de cultura”. Essa visão dinâmica da cultura do mediterrâneo antigo afasta-se de uma ideia de linearidade, abarcando as porosidades de um tempo histórico complexo. Tal abordagem acerca do passado greco-romano se ancora nos estudos do hamburguês Aby Warburg (1866-1929) em torno da “sobrevivência da Antiguidade” [*Nachleben der Antike*]: as expressões gregas e romanas atuam como uma força em movimento na memória cultural da humanidade, tendendo a retornos metamorfoseados num dado contexto geográfico e temporal. Warburg (2013, p.475-476) é explícito ao enfatizar as possíveis relações entre passado antigo e presente moderno para além de categorias fechadas em si, onde podem se considerar “a Antiguidade, a Idade Média e a Idade Moderna como épocas interrelacionadas”.

O atravessamento temporal da *Nachleben* gravita em torno da dimensão simbólica das imagens na prática histórico-cultural, pois estas, em si, são forças plásticas, submetidas a transformações conforme suas migrações. Do ponto de vista da produção visual da *Eu Sei Tudo*, a inserção da Antiguidade pagã subsistiu na construção paradoxal da imagem da mulher moderna pela força associativa dos atributos das divindades antigas. Entremeando o aspecto guerreiro e republicano de Minerva e Vesta, a pureza virginal e doméstica das vestais ressurge em diálogo com a expressão da beleza pela Juno romana: motivo, tema e forma associaram a deusa antiga à mulher do século XX, numa descontinuidade temporal que criava algo novo. As capas e propagandas da *Eu Sei Tudo* analisadas aqui caracterizam, portanto, uma montagem de tempos: o sentido da montagem se baseia na criação de um grande sistema figurativo de expressões visuais (Didi-Huberman, 2013, p.407) na revista.

Na *Eu Sei Tudo*, a associação entre a imagem desterritorializada com a palavra local evidencia uma determinada expressividade das deusas pagãs. Essa expressividade foi conceituada por Warburg (2013, p.285-286) como “fórmulas de *páthos*” [*Pathosformenl*]: modelos ou motivos da Antiguidade que sobrevivem e são instrumentalizados para a expressão da intensificação emotiva interna na gestualidade corporal. Tal quadro nos permite reconhecer a *Nachleben* de uma deusa que foi identificada por Warburg em todos os seus estudos: a ninfa. Na Antiguidade, as ninfas eram divindades das águas claras, das fontes e das nascentes, além de expressar visualmente os elementos femininos por excelência (Agamben, 2010, p.10). Descrita por Warburg como uma “deusa pagã no exílio”, a revisitação da ninfa representa a inserção dos motivos de movimento intensificado da Antiguidade sobrevivente nas expressões e indumentárias das personagens femininas (Campos, 2020, p.227).

O retorno da ninfa pode ser identificado nas montagens das capas da *Eu Sei Tudo*: os atributos divinos conferiram uma mimética que caracteriza as principais virtudes da mulher moderna associadas ao paganismo sobrevivente. Ao erguer o fogo da vestal, típico da pacificação da pátria e da pureza virginal para a mulher do pós-guerra; do *páthos* triunfal da vitória guerreira, ao ostentar os louros do progresso; e a quebra da placidez pelo movimento do tecido como ferramenta do patético que confere a magnificência de uma ninfa moderna pelos atributos de Juno (Didi-Huberman, 2002, p.21). A ninfa, portanto, incorpora uma psicologia histórica da expressão de um ideário feminino por meio desse retorno ao antigo, que tem seu sentido restituído e ampliado pela operação da montagem no *magazine*.

## As imagens do Antigo nas propagandas e a mulher greco-romana na modernidade brasileira

Partindo da leitura da revista ilustrada como portadora de um determinado discurso visual para a vida moderna construído com referências na Antiguidade, identificamos a presença de numerosas divindades greco-romanas em propagandas na *Eu Sei Tudo*. As revistas do século XX foram alguns dos principais suportes de difusão das práticas higienistas nas cidades brasileiras, associadas à lógica de modernização e progresso por meio de propagandas de consumo de medicamentos. O corpo se tornou um espaço de investimento, normatização e correção pelo discurso biomédico: a propaganda argumentava não apenas que a enfermidade era algo passível de correção por meio do fármaco, mas que o ato de adquirir o medicamento fazia com que o consumidor incorporasse a modernidade que o avanço da ciência representava (Heine, 2014, p.6).

Os anúncios de produtos farmacêuticos, relacionados à higiene e à saúde – principalmente das mulheres – penetraram as páginas das revistas ilustradas com maior vigor a partir da década de 1940, quando o mercado de bens de consumo e a profissionalização da imprensa tornaram-se peças-chave para a circulação das propagandas de produtos fornecidos pelo parque industrial (Kobayashi, 2018, p.744). Alinhada a esse processo estava a ideia de que a higiene poderia ser adquirida a partir do uso de sabonetes para a prevenção de maus odores e de que a saúde do indivíduo poderia ser rejuvenescida com o uso de medicamentos. As propagandas, portanto, tinham como finalidade “persuadir o consumidor de que essas aquisições representariam um investimento na vida pessoal, na manutenção da saúde e no bem-estar da família” (Hochman, Kobayashi, 2015, p.68).

Na *Eu Sei Tudo*, as propagandas de medicamentos se concentravam, na maioria das edições, no início ou ao final da revista. No presente estudo, serão analisadas as propagandas de dois medicamentos divulgados no período de circulação da revista: Bayer e Vikelp, abrangendo o período de 1919 a 1945.

Nas propagandas da Bayer analisadas na *Eu Sei Tudo*, o principal medicamento recorrente para incentivo ao consumo era o comprimido de aspirina, podendo ser, ocasionalmente, apresentado na modalidade de cafiaspirina (quando combinados o ácido acetilsalicílico e o agente potencializador da cafeína).^5^ A medicação tinha atuação analgésica e anti-inflamatória para combater dores de cabeça moderadas e fortes, sendo indicada para pessoas com recorrência de enxaquecas e vendido em tubos de vinte a trinta comprimidos.^6^

**Figura 4:**
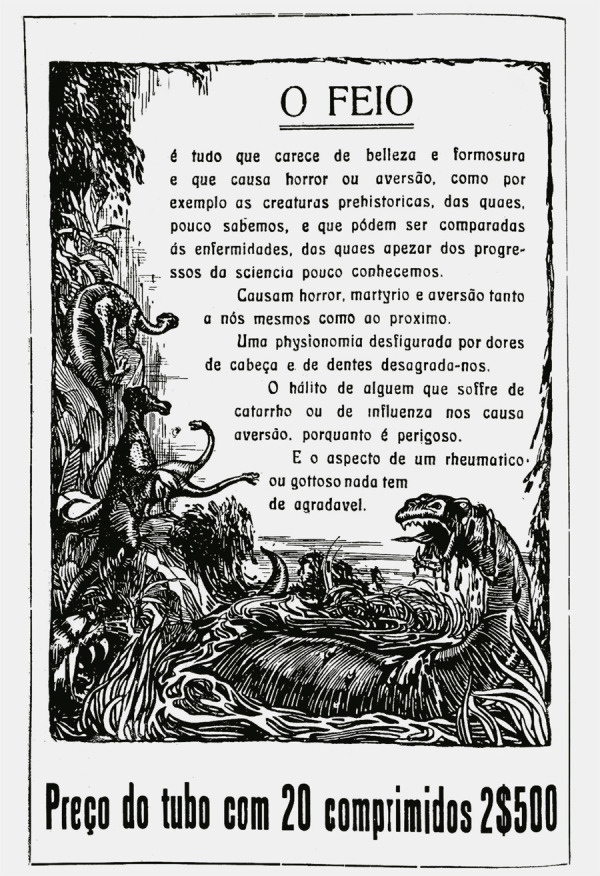
: Propaganda de aspirina da Bayer (Eu Sei Tudo..., jun. 1919, p.150)

O anúncio da aspirina presente na edição de junho de 1919 da *Eu Sei Tudo* seguiu um determinado padrão de associação entre escrita e imagem: dois textos complementares distribuídos numa página dupla, emoldurados por borda decorativa estilizada que atuava como fator de integração. Assim, a propaganda se estendia pelas duas páginas, numa leitura de oposição e complementaridade (Figuras 4 e 5). O texto começava na página par, com o título “O Feio” sendo descrito como algo “que causa horror ou aversão”. Tal descrição é associada aos males da enxaqueca, como imagem negativa para o consumidor: “Uma fisionomia desfigurada por dores de cabeça e de dentes desagrada-nos” (Eu Sei Tudo..., jun. 1919, p.150). Identifica-se a construção visual da dor de cabeça como algo primitivo e selvagem, a partir da estetização de uma selva pré-histórica, com destaque para imagens de vegetações em decomposição, animais peçonhentos e desconhecidos emergindo de um lodaçal, numa tonalidade escura dominando a composição.

**Figura 5:**
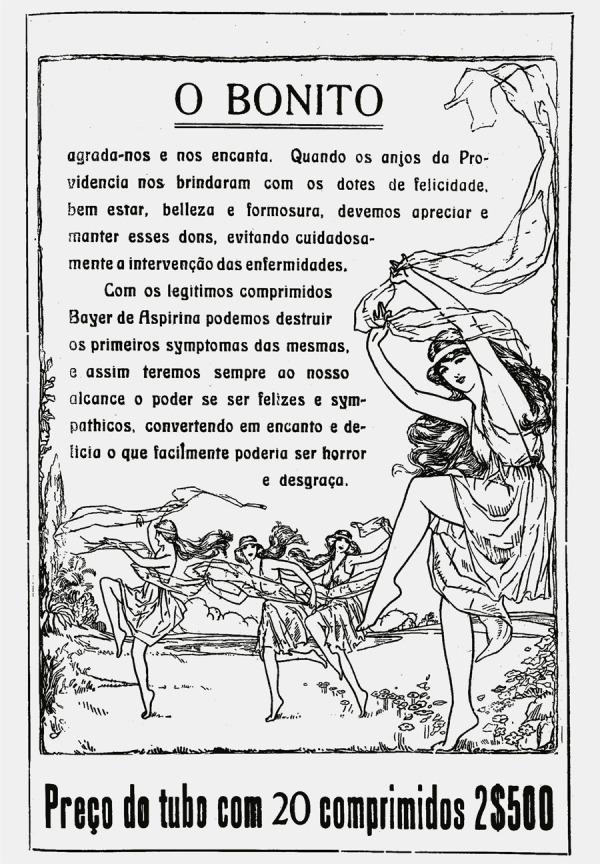
: Propaganda de aspirina da Bayer (Eu Sei Tudo..., jun. 1919, p.151)

Na página ímpar, um novo texto continua a propaganda, com as orientações das formas para curar o mal citado a partir do consumo da medicação. Intitulada “O Bonito”, faz oposição à página anterior, pois “agrada-nos e nos encanta”. Em seguida, define-se que o “bonito” só é alcançado por meio de um ideal de equilíbrio e encanto, a partir de uma origem “divina” que só pode ser mantida com o consumo da aspirina:

Quando os anjos da providência nos brindaram com os dotes de felicidade, bem-estar, beleza e formosura, devemos apreciar e manter esses dons, evitando cuidadosamente a intervenção das enfermidades.

Com os legítimos comprimidos de Bayer de Aspirina podemos destruir os primeiros sintomas das mesmas, e assim teremos sempre ao nosso alcance o poder de ser felizes e simpáticos, convertendo em encanto e alegria o que poderia ser horror e desgraça (Eu Sei Tudo..., jun. 1919, p.151).

Percebemos como a matriz do discurso biomédico se entrelaça a uma perspectiva de felicidade e ideal de beleza inata, a partir da marca Bayer de aspirina. Especificamente, aponta-se que as moléstias que os comprimidos podem evitar têm a função de manter aspectos que extravasam o interior, abrangendo o comportamento, o humor e a aparência. Identificamos a associação direta com a ilustração da borda decorativa: quatro figuras femininas se apresentam num ritmo de dança, envolvidas por um tecido contínuo, acompanhando e integrando seus movimentos corporais. Ao fundo, o ambiente é florido, aproximando-se de uma imagem paradisíaca, que reforça a fluidez corporal das mulheres numa aura de delicadeza. Pela composição visual, pode-se indicar que as quatro figuras femininas são as alegorias representativas da felicidade, bem-estar, beleza e formosura, elencadas na propaganda como valores divinos.

Pelos movimentos nos cabelos e trajes, percebe-se a inserção dos modelos antigos no drapeado dos corpos femininos, à maneira das ninfas dançantes sobreviventes. Ainda que no texto da propaganda fosse projetada a ideia de que a medicação estivesse atrelada a uma calmaria idealizadora, a adoção das alegorias evidencia um retorno recalcado de um frenesi pagão para expressão de valores da saúde para a mulher na modernidade. Ao debruçar-se sobre a ninfa como personagem teórica, Campos (2020, p.243) aponta que a imagem encarna a *Nachleben* por meio da relação entre a rememoração antiga e uma nova vida, evidenciando como sua figuração atravessa a história para promover a inserção do movimento como expressão do feminino. Isso faz eco às reflexões de Didi-Huberman (2013, p.220) ao ressaltar o caráter dialético da ninfa sobrevivente, que oscilava entre a imagem da deusa e da mulher, encarnando a *Pathosformel* feminina: “Vênus terrestre e Vênus celeste, dançarina e Diana, serva e Vitória, Judite castradora e Anjo feminino”. Considerando o modo como as propagandas se pautam na lógica de consumo da vida moderna, percebe-se como o olhar sobre tais imagens propicia o desvelamento da sobredeterminação temporal da imagem ao reportá-la a outros regimes visuais marcados por configurações anacrônicas a partir da inserção do antigo.

**Figura 6:**
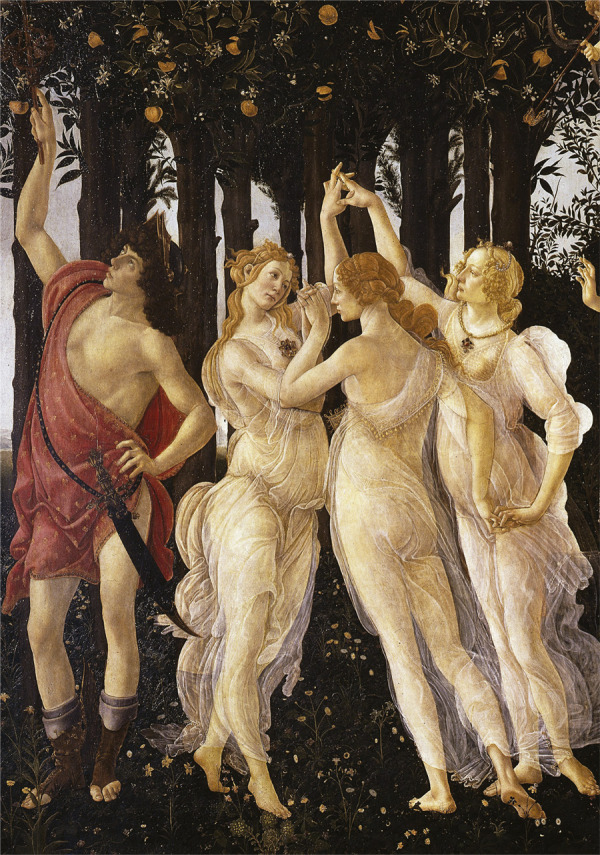
: *A Primavera*, detalhe com as Graças e Mercúrio, de Sandro Boticelli, têmpera sobre madeira, 1482 (Google Arts & Culture)

Remetendo aos repertórios de imagens sobreviventes, a dinâmica da ninfa desterritorializada atravessa o “anjo” da aspirina*,* na reaparição do componente patético: o olhar que examina a dança da boa saúde nas ninfas da Bayer permite associá-las com o ritmo das “três graças”, figuras míticas que simbolizam a elegância feminina e os benefícios.^7^ Segundo a tradição literária e artística romana, encontram-se posicionadas ao lado de Mercúrio^8^ (Borges, Silveira, 2019, p.125) ou próximas a divindades da medicina como Esculápio^9^ (Ménard, 1985, p.292). Warburg (2015, p.27-86) identificou a ninfa sobrevivente nas pinturas renascentistas, como *A Primavera*, de Sandro Botticelli (Figura 6): ela irrompe na excitação corporal do tecido das Graças que atravessa o tempo e espaço para animar o anjo da medicina moderna na revista. O retorno do movimento da ninfa/graça foi marcado, também, pela estética do início do século: a mulher antiga era também moderna pelas formas sinuosas do *art nouveau* que estilizou a propaganda.

Na década de 1940, o enfoque nos padrões de beleza para a mulher moderna dominou as páginas da *Eu Sei Tudo*, tendo grande espaço nas peças publicitárias da Vikelp. O comprimido Vikelp era uma medicação que tinha a finalidade de combater o complexo de magreza, tido como indesejado e objetivando o consumo para mulheres que desejassem ter uma compleição física robusta e curvilínea como um caminho para a felicidade e realização pessoal (Heine, 2014, p.11). Segundo Nardini et al. (2018, p.9), os anúncios da Vikelp utilizavam imagens de mulheres com roupas que valorizassem o corpo com curvas, como trajes de banho, e poderiam vir acompanhados de uma ilustração. Nas edições da *Eu Sei Tudo* que circularam em março e setembro de 1945, as propagandas ofereciam “O vigor e a perfeição do corpo humano” em oposição à mulher “magra, debilitada e nervosa”, fornecendo suplementação vitamínica a base de iodo para transformar o corpo magro em “carnes rijas” (Eu Sei Tudo..., mar. 1945, p.104).

As imagens dos anúncios imbricavam espaço e tempo para criar o ideal de corpo saudável propagado pela Vikelp: na *Eu Sei Tudo* de março, a mulher moderna esbanja esbelteza corporal numa inclinação em paralelo com um desenho da estátua da *Vênus de Milo*.^10^ Alçada como padrão de beleza natural para a modernidade, a Vênus de Milo evocava a imagem da heroína: suas formas corporais se apresentam impregnadas do vigor vitorioso da abundância, representativa da capacidade de gerar e nutrir, atributos considerados fundamentais para o corpo feminino (Ménard, 1985, p.275). Aspecto similar pode ser encontrado na propaganda da Vikelp de setembro do mesmo ano, em que a mulher ideal é apresentada em seu maiô ao lado da *Diana de Versalhes* ^11^ (Eu Sei Tudo..., set. 1945, p.103). Conhecida como deusa da natureza, Diana (associada à Ártemis grega) é a caçadora por excelência e senhora das feras, além de protetora dos partos e dos aspectos virginais, regendo as ninfas dos bosques (Chevalier, Gheerbrant, 2020, p.130). No mármore, a deusa coroada com o diadema é retratada num movimento de corrida acompanhada pela corça de Cerineia.^12^ A dinâmica é ditada pelo ritmo da túnica pregueada e na inclinação corporal, ao levar a mão direita à aljava de flechas e a esquerda sobre um arco, hoje inexistente na composição. Apontamos anteriormente (Maia, Oliveira, 2020, p.61) que a imagem da deusa caçadora seria a personificação do corpo sadio a ser alcançado pela medicalização proposta pela Vikelp, em paralelo à esbelta mulher do século XX.

**Figura 7:**
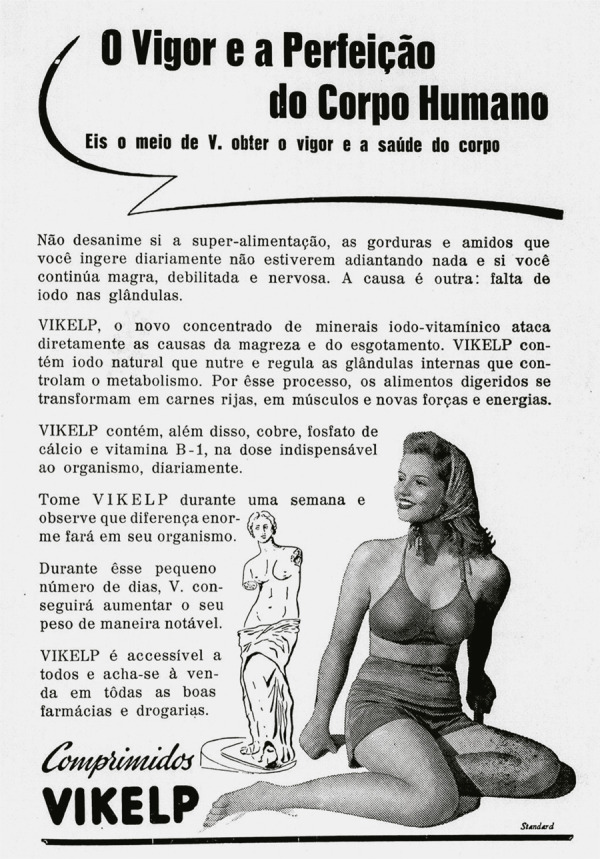
: Anúncio de comprimidos da Vikelp (Eu Sei Tudo..., mar. 1945, p.104)

**Figura 8:**
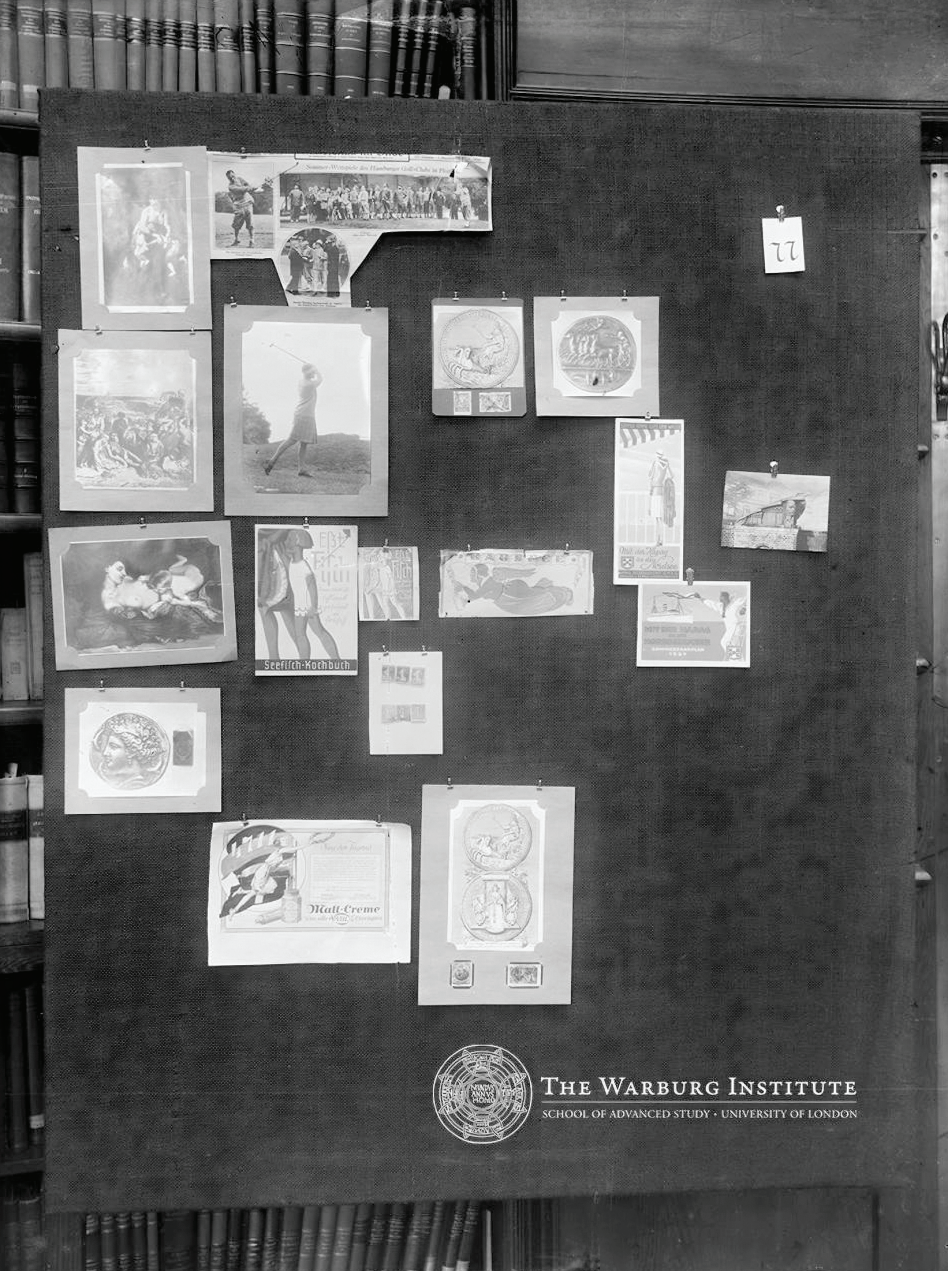
: Prancha 77 do *Bilderatlas Mnemosyne* (The Warburg Institute)

Segundo analisamos àquela altura, o destaque para a correlação entre antigo e moderno se dá, justamente, no movimento corporal que aproxima a obra de arte da mulher do cotidiano, caracterizando a *Nachleben* da ninfa. A propaganda como uma experimentação de montagem visual se aproxima da proposta do *Bilderatlas Mnemosyne* [Atlas de Imagens Mnemosyne], idealizado por Warburg, mas inacabado por ocasião de seu falecimento. Considerado por Didi-Huberman (2013, p.383) um pensamento por imagens, o *Atlas* era composto por pranchas que produziam uma série comparativa de formas da vida em movimento em suportes e tempos distintos. No que tange à sobrevivência da ninfa, a prancha de número 77 se torna a mais expressiva ao centralizar a fotografia de uma golfista num descompasso que a aproxima da deusa. Nas palavras de Campos (2020, p.240), a golfista é “uma heroína olímpica que parece imitar o gesto do Olimpo”. Com a mesma energia, a ninfa exilada encontra nova roupagem na propaganda da Vikelp: mantém a tensão de retorno aos modelos da beleza e da castidade antigos, mas encarnados na nova mulher, que deve almejar um corpo saudável, forte e útil para a atividade do lar e do mercado de trabalho (Maia, 2012) (Figuras 7, 8 e 9).

**Figura 9:**
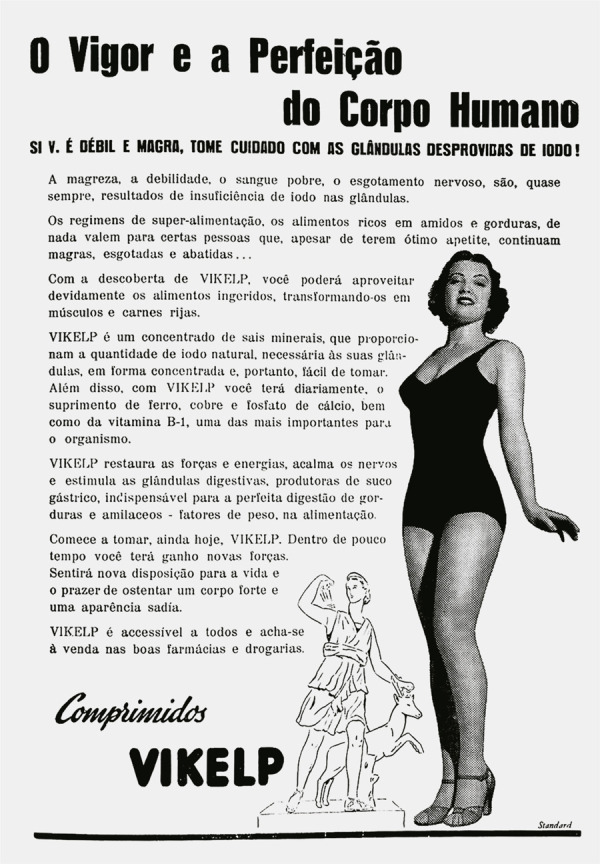
: Anúncio de comprimidos da Vikelp (Eu Sei Tudo..., set. 1945, p.103)

## Considerações finais: A Cinta Moderna

Ao abordar a presença da ninfa na modernidade, Didi-Huberman (2002, p.136) considerou que “se encontra lançada uma hipótese sobre a necessidade coletiva, cultural, de uma sobrevivência moderna dos deuses pagãos em geral”. Entremeando Vênus, Diana e as graças, a ninfa aporta na propaganda brasileira da primeira metade do século XX como uma autêntica *Nachleben der Antike*, onde suas variadas figurações pelas *Pathosformenl* permitem uma revisitação sobre as formas de expressão que quebram hierarquias temporais. O modelo dos anúncios em suportes de ampla circulação apresenta, a partir da análise da ninfa, um retorno ao antigo atravessado pelo imperativo da vida moderna idealizada pelo corpo feminino saudável gerado pelo olhar do consumidor. No percurso pelas propagandas da Bayer e da Vikelp, a imagem antiquizante compõe a temática do discurso biomédico no qual a representação dos valores higienistas modernos é realizada pela presença das divindades desterritorializadas. Do mundo greco-romano vinha a referência cultural, que pela colisão de tempos e retornos com a cultura técnica moderna tornou a peça publicitária um espaço de montagem para alcançar o olhar e a prática da vida.

**Figura 10:**
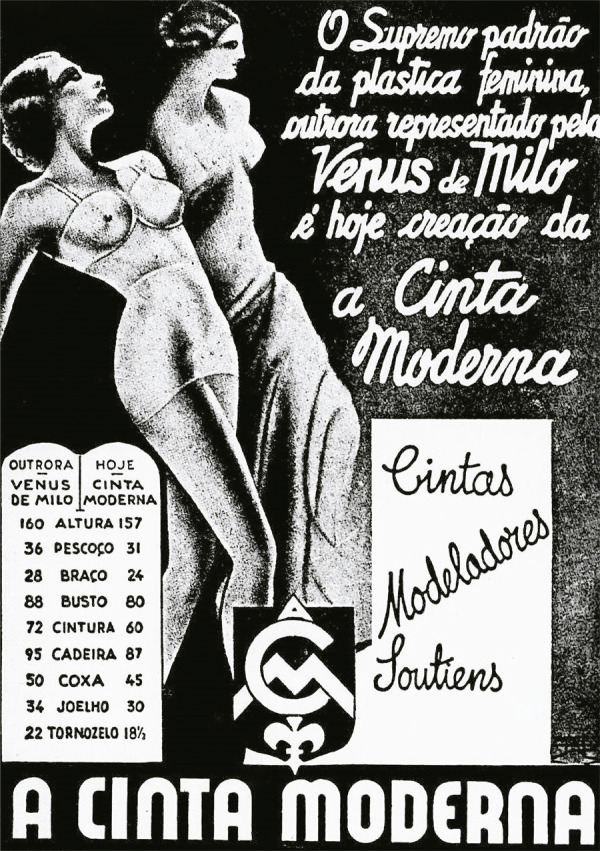
: Anúncio de A Cinta Moderna (Eu Sei Tudo..., dez. 1941, p.85)

Essa relação associativa entre palavra e imagem nas propagandas, portanto, aproxima o perfil das revistas ilustradas da proposta de exposição visual de descontinuidades propiciada pelo *Bilderatlas Mnemosyne* idealizado por Warburg. Na quebra da fronteira temporal, geográfica e formal, a ninfa antiga emerge como a mulher moderna pela tensão e imbricação: uma das imagens mais emblemáticas dessa montagem pode ser encontrada na propaganda de A Cinta Moderna, presente na edição de dezembro de 1941 da *Eu Sei Tudo* (Figura 10). Criada em 1927, A Cinta Moderna integrou o contexto da produção de *lingeries* que se adequassem à modelagem natural do corpo feminino, abandonando as barbatanas rígidas e se adaptando a um novo ideal de educação física da mulher (Fields, 2007).

Na propaganda, as heterogeneidades de tempos são animadas a partir de uma configuração de significações, gerando algo novo pela potência visual criada pela interrelação entre título e imagem. Eis a “fusão” do ideal de beleza antigo na mulher brasileira: a revolução técnica de A Cinta Moderna só foi possível pelo seu retorno à Vênus de Milo, ao propiciar “o supremo padrão da plástica feminina” para todas. “Outrora – Vênus de Milo” e “Hoje – Cinta Moderna”, inseridas numa tábua romana adornada com uma grafia moderna e decorada com a flor-de-lis francesa. Eis os ornamentos, detalhes e inclinações em que pulsa a sobrevivência da ninfa. O panteão greco-romano permanece como modelo cultural que transcende fronteiras.

## Data Availability

Não estão em repositório.

## NOTAS

1 A presença da Antiguidade greco-romana permeia o cotidiano do Rio de Janeiro em obras arquitetônicas que compõem a paisagem urbanística da cidade. Dentre essas, destaca-se o Palácio Tiradentes, inaugurado em 1926, adornado com esculturas que alegorizam o progresso republicano a partir de figuras de deuses da Antiguidade.

2 Seth foi um pioneiro do desenho animado no Brasil, destacando-se pela crítica social e política. Nas primeiras décadas do século XX integrou um importante grupo – com K. Lixto e J. Carlos – que atuou na transformação da produção gráfica das revistas cariocas como modo de performar a modernidade.

3 Na sociedade romana, a deusa Vesta era a personificação do fogo sagrado do lar e da cidade, correspondendo à deusa Héstia grega.

4 A deusa romana Victoria personificava a vitória na Antiguidade romana, associada aos ideais de *honor et gloria* [honra e glória]. Sua origem remete à Niké grega, filha de Palas e Estige, representada por uma mulher alada e portadora de louros, pronta a coroar os heróis e vitoriosos.

5 A Chimica Bayer Ltda, sucursal da Bayer no Brasil, fez parte do Instituto Behring de Terapêutica Experimental, criado em 1932, no Rio de Janeiro. A empresa auxiliou na promoção dos produtos farmacêuticos alemães no mercado brasileiro, utilizando-se amplamente da propaganda científica em revistas ilustradas.

6 A aspirina foi um dos grandes produtos farmacêuticos comercializados pela Bayer no Brasil. Dados apresentados por Quintaneiro (2002, p.153) demonstram que entre 1935 e 1939 produziu-se no Brasil cerca de 178 milhões de comprimidos de cafiaspirina. O mercado sul-americano era um dos mais lucrativos.

7 As graças eram figuras míticas descendentes de Júpiter que simbolizavam a graça e a beleza femininas, sendo associadas ao séquito de Vênus ou de Apolo. Ainda que fossem imortais, não tinham origem divina. Tradicionalmente são três: Aglaia (“Esplendor”), Talia (“Florescência) e Eufrósina (“Alegria”).

8 O deus Mercúrio (associado ao Hermes no mundo grego) era o deus do comércio, das viagens e das passagens entre os dois mundos. Seus principais atributos são o caduceu (uma vareta alada onde se entrecruzam duas serpentes) e o pétaso (um chapéu com pequenas asas que imprimiam a imagem da velocidade do mensageiro).

9 Filho de Apolo e Corônis, Esculápio era o deus da medicina e da saúde. A serpente lhe foi atribuída, tendo-se tornado o emblema da medicina. Equivale ao deus Asclépio no mundo grego.

10 A escultura da deusa Vênus de Milo tem sua provável origem no século II a.C., atribuída ao grego Alexandre de Antioquia. Feita em mármore de Paros, foi redescoberta na Grécia do Império Otomano em 1820, tendo sido adquirida pela França e se notabilizado como uma das principais obras do Museu do Louvre.

11 Atribuída ao grego Leócares, o mármore da *Diana de Versalhes* tem sua provável datação no século IV a.C. Também chamada de *Artemísia, deusa da caça*, foi oferecida à França no século XVI, tendo passado a integrar o Louvre a partir de 1789.

12 Animal consagrado a Diana, tinha chifres de ouro, pés de bronze e era dotada de grande velocidade. Foi capturada por Héracles em sua terceira tarefa.
